# Assessing the risk of early unplanned rehospitalisation in preterm babies: EPIPAGE 2 study

**DOI:** 10.1186/s12887-019-1827-6

**Published:** 2019-11-21

**Authors:** Robert Anthony Reed, Andrei Scott Morgan, Jennifer Zeitlin, Pierre-Henri Jarreau, Héloïse Torchin, Véronique Pierrat, Pierre-Yves Ancel, Babak Khoshnood

**Affiliations:** 1 Université de Paris, Epidemiology and Statistics Research Center/CRESS, INSERM, INRA, F-75004 Paris, France; 2 Elizabeth Garrett Anderson Institute for Womens’ Health, UCL, London, UK; 3 SAMU 93, SMUR Pédiatrique, CHI André Gregoire, Groupe Hospitalier Universitaire Paris Seine-Saint-Denis, Assistance Publique des Hôpitaux de Paris, Paris, France; 4 APHP.5, Service de Médecine et Réanimation Néonatales de Port-Royal, Paris, France; 5 Department of Neonatal Medicine, CHU Lille, Jeanne de Flandre, Lille, France; 6 Clinical Research Unit, Center for Clinical Investigation P1419, APHP.5, F-75014 Paris, France

**Keywords:** Prematurity, Newborn, Neonatology, Rehospitalisation, Discharge, Prediction, Survival analysis, Epidemiology, Cohort study

## Abstract

**Background:**

Gaining a better understanding of the probability, timing and prediction of rehospitalisation amongst preterm babies could help improve outcomes. There is limited research addressing these topics amongst extremely and very preterm babies. In this context, unplanned rehospitalisations constitute an important, potentially modifiable adverse event. We aimed to establish the probability, time-distribution and predictability of unplanned rehospitalisation within 30 days of discharge in a population of French preterm babies.

**Methods:**

This study used data from EPIPAGE 2, a population-based prospective study of French preterm babies. Only those babies discharged home alive and whose parents responded to the one-year survey were eligible for inclusion in our study. For Kaplan-Meier analysis, the outcome was unplanned rehospitalisation censored at 30 days. For predictive modelling, the outcome was binary, recording unplanned rehospitalisation within 30 days of discharge. Predictors included routine clinical variables selected based on expert opinion.

**Results:**

Of 3841 eligible babies, 350 (9.1, 95% CI 8.2–10.1) experienced an unplanned rehospitalisation within 30 days. The probability of rehospitalisation progressed at a consistent rate over the 30 days. There were significant differences in rehospitalisation probability by gestational age. The cross-validated performance of a ten predictor model demonstrated low discrimination and calibration. The area under the receiver operating characteristic curve was 0.62 (95% CI 0.59–0.65).

**Conclusions:**

Unplanned rehospitalisation within 30 days of discharge was infrequent and the probability of rehospitalisation progressed at a consistent rate. Lower gestational age increased the probability of rehospitalisation. Predictive models comprised of clinically important variables had limited predictive ability.

## Background

Preterm births affect approximately 9% of live births in Europe [1], and have substantial repercussions for a newborns’ short and long term health outcomes, as well as for health systems and wider society [2–4]. In this context, unplanned rehospitalisations can be useful markers for serious pathologies, and also represent potentially modifiable adverse events. Preventing unplanned, and in some cases avoidable, rehospitalisations can potentially reduce costs, the risk of iatrogenic effects and wider burdens on babies and their families. A better understanding of rehospitalisation rates, timing and predictive models aiming to provide objective estimates of a preterm baby’s risk could potentially complement clinical judgment and inform decision making [5–9].

Limiting rehospitalisations is a key challenge facing health systems as they are associated with large costs and inconvenience to patients and providers [10–12]. Thirty-day rehospitalisations are a particular focus for health providers [13–15]. Rehospitalisation rates amongst preterms have been found to be significantly higher than those of full-term infants [16–18]. Factors previously found to be associated with rehospitalisation are male sex [19–22], lower gestational age [23–25], low birth weight or being small for gestational age (SGA) [26], feeding problems [27–30], bronchopulmonary dysplasia (BPD) [31, 32] and lower socioeconomic status [21, 27, 33, 34]. To the best of our knowledge, the literature on the early rehospitalisation of preterms discussed explanatory models only and not validated predictive models. As explanatory models often do not provide optimal predictions, the literature cannot directly address one of our aims of predicting rehospitalisations [35–37].

In this study, using data from a large, prospective, population-based cohort study of newborns of 22–34 weeks gestation (EPIPAGE 2) [38] we examined early (≤30 days) unplanned rehospitalisations following initial discharge. Our first objective was to look at the probability and timing of unplanned rehospitalisations during the 30-day period following initial discharge. Our second objective was to assess the ability of a set of important clinically relevant variables to predict unplanned rehospitalisation within 30 days of discharge.

## Methods

### Study design and population

**Fig. 1:**
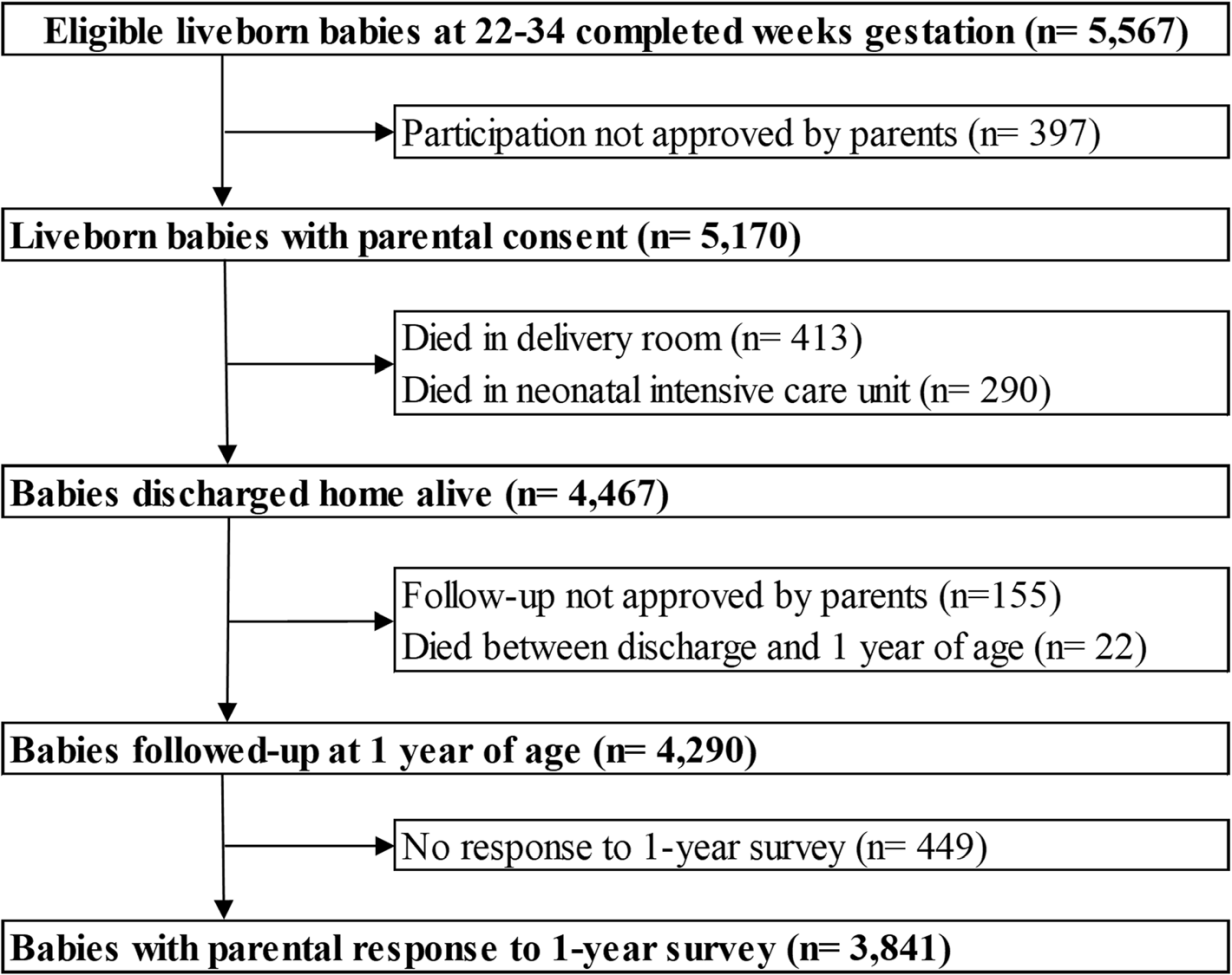
Flow chart of the study population derived from the EPIPAGE 2 cohort

This study used data from the EPIPAGE 2 cohort, a French national prospective study. The eligible population of EPIPAGE 2 included all babies born at 22–34 weeks gestation in all maternity units in 25 regions of France. The study began on March 28, 2011, and ended on December 31, 2011 and recruited from all maternity units in participating regions. The one region that did not participate in the study accounted for just over 18,000 births in 2011, around 2% of all births in France. Babies with a gestational age of 22–26, 27–31 and 32–34 weeks had recruitment periods of 8 months, 6 months and 5 weeks respectively [38]. All babies discharged home alive following birth hospitalisation and whose parents completed the one-year survey were eligible for inclusion in our study. Babies who died during the initial birth hospitalisation or between discharge and one-year follow-up were excluded. Babies of parents that either did not consent to, or failed to complete, the one-year follow-up survey were also excluded. A flow chart of the selection of the study population can be seen in Fig. 1.

The EPIPAGE 2 study collected data at birth and at follow-up at one, two and 5.5 years corrected age. We used data collected at birth and the one-year follow-up. Birth data were collected during the neonatal period in maternity and neonatal units using medical records and questionnaires for obstetric and neonatal teams. Neonatal data collection addressed the baby’s birth condition, disease status and treatments received. Interviews and self-administered questionnaires were used in the neonatal unit to obtain information on a mother’s socioeconomic status, health and the care her baby received prior to discharge. The one-year follow-up survey comprised a questionnaire sent to the parents to collect details of post-neonatal care, hospitalisations, growth, sequela, treatments, maternal health, and socioeconomic details.

### Outcome

We looked at both the overall probability of early (≤30 days) unplanned rehospitalisations and the timing of unplanned rehospitalisations during the 30 days following initial discharge of newborns from the hospital. Unplanned rehospitalisation status (URH) was defined according to the recorded cause of rehospitalisation. This information was collected from parents via the one-year follow-up survey requesting the date and cause of their baby’s three longest rehospitalisations. For cause, parents could select from bronchiolitis or asthmatic bronchitis, gastroenteritis, diarrhoea or dehydration, poor weight gain, convulsion, injury, malaise, surgery or other (‘vaccination’ or ‘for observation’ for example). Rehospitalisations for surgery and vaccinations were classified as planned, all other causes were considered unplanned. Any baby with both an unplanned cause (e.g. gastroenteritis) and planned cause (e.g. surgery) of rehospitalisation was classified as having a URH. The number of days between initial discharge and the first URH was used to determine whether a baby had an URH within 30 days of initial discharge (URH30). In cases where a baby had multiple URH30 the earliest of these was selected and used in analysis.

### Predictor variables

A model containing the predictor gestational age alone was constructed initially to provide a performance baseline. Following a review of the literature and discussion with expert clinicians, 48 predictors (Additional file 1) were then considered for inclusion in a ten predictor and 20 predictor model. Selection was informed by exploratory analysis, cross tabulation, consideration of a variable’s reliable availability and further discussion with clinicians. For the ten predictor model, emphasis was placed on selecting a parsimonious model, potentially practical for clinical use. The ten predictor model contained: sex (binary), gestational age in weeks (categorical; 22–26, 27–31 and 32–34), SGA status (binary; weight below the 10th percentile for gestational age), exposure to nitric oxide (binary), surfactant (binary), bronchopulmonary dysplasia (BPD) (categorical; none, mild (≥28 days oxygen and breathing room air to week 36), moderate (≥28 days oxygen and mechanical ventilation or continuous airway pressure/ FiO_2_ > 21% at week 36) or severe (≥28 days oxygen and mechanical ventilation or continuous airway pressure/ FiO_2_ > 30% at week 36)), early onset neonatal infection (binary; no infection or either a probable infection with antibiotics started before 72 h of life and duration ≥5 day or certain infection with positive blood or cerebrospinal fluid culture before 72 h of life), post-menstrual age at discharge (PMA) in weeks (four categories of approximately equal size; <36, 36- < 37, 37- < 38 and ≥ 38), discharge weight in grams (four categories of approximately equal size; ≤2200, 2201-2600, 2601–3000 and > 3000) and breastfeeding status at discharge (categorical; recording whether baby was receiving either no breast milk, mixed feeding or exclusive breastfeeding at discharge).

To investigate the impact of model complexity and the influence of wider clinical, maternal and socioeconomic factors on prediction, a 20 variable model comprised of all predictors from the ten predictor model plus an additional ten was developed. The additional predictors were: multiple pregnancy (binary), level of birth unit (categorical; 1, 2a, 2b or 3), congenital abnormalities (binary), late onset neonatal infection after >72 h of life (binary), necrotising enterocolitis (binary), intraventricular hemorrhage (IVH) (Stage 3 IVH or intraparenchymal hemorrhage) (binary), mother’s age in years (continuous), mother born outside France (binary), family socioeconomic status (categorical; professional, intermediate, administrative/public service, self-employed/students, shop assistants, service workers, manual workers or no profession) and smoking during pregnancy (binary).

### Statistical analysis

We compared the characteristics of babies with URH30 to those without, using the Kruskal-Wallis test for continuous variables and chi-squared test or Fisher’s exact test for categorical variables.

To investigate the timing and proportion of babies with URH over the first 30 days we produced Kaplan-Meier curves for URH status censored at 30 days alone and also according to gestational age category. Ninety-five percent confidence intervals and the log-rank test were used to establish whether there were any differences between Kaplan-Meier curves.

A *p* value of <0.05 was considered statistically significant. All analysis was conducted using R version 3.4.2 [39].

#### Predictive model building and validation

Three predictive models for URH30 were constructed using complete-cases (babies with no missing values for the outcome or exposure). The first model contained the gestational age predictor alone, the second contained ten predictors and the third model contained 20. Multivariate logistic regression analysis was used to construct the models. The performance of each model was validated with 10-fold cross-validation [40, 41]. This involved dividing the complete-cases into ten equally sized subsets. Each time, nine of the subsets were used to train an independent regression model. The coefficients derived from the training stage were then used to predict URH30 on the one remaining test subset. This was repeated ten times until each subset had been used as the test once. Coefficients for the model were derived by training the model on the entire data set as the performance measured with the cross-validation subsets is assumed to be an approximation of the performance of the model trained on all samples [42]. Model performance was assessed using measures of discrimination and calibration. Discrimination was measured using area under the receiver operating characteristic curve (AUROC) with 95% confidence interval, sensitivity, specificity and Tjur’s coefficient of determination [43]. Calibration was assessed via the Hosmer-Lemeshow goodness-of-fit test and corresponding calibration curve. The classification threshold for predictive modelling was adjusted to optimise the false positive and true positive rates [44–46]. The impact of the categorisation of continuous variables such as gestational age and birth weight on prediction was assessed through a sensitivity analysis for the predictive models.

#### Missing data

To establish the impact of missing data upon prediction we used multiple imputation and rebuilt the predictive models using the imputed data [47]. Imputation was conducted using 20 imputations and 100 iterations. The full list of variables used as predictors during imputation and their rate of missingness can be seen in Additional file 2. Pooled model coefficients were derived using Rubin’s rule [48] and performance measures presented using the median and inter-quartile range [49–51].

## Results

There were 5567 live born babies eligible for inclusion in the EPIPAGE 2 study; 703 died during the initial hospitalisation and 4467 were discharged home alive, of these, 3841 babies (86% of those babies discharged home alive) had parents that completed the one-year follow-up survey (Fig. 1). Compared to the 3841 babies included in our eligible population, those that were excluded due to their parents not completing the one-year follow-up survey (449 babies) had significantly higher median birth weight (1410 g v 1350 g, *p* = 0.040) and levels of maternal unemployment (9.6% v 2.3%, *p* < 0.001). Median maternal age was also lower compared to the eligible population (28 years v 30 years, *p* < 0.001), and there were lower levels of exclusive breastfeeding amongst babies excluded for lack of survey completion (18.4% v 28.2%, *p* < 0.001). Babies that died between discharge and the one-year follow-up, and were therefore excluded from the analysis, had lower levels of exclusive breastfeeding compared to the eligible population (9.0% v 28.2%, *p =* 0.047). They also had higher rates of severe BPD (18.0% v 6.0%, *p =* 0.017).

There were 399 30-day rehospitalisations for any cause in our sample, a rate of 10.4% (95% CI 9.4–11.4). Three hundred and fifty (9.1% (95% CI 8.2–10.1)) newborns in the study population experienced an unplanned rehospitalisation within 30 days of index discharge. A proportion of these URH30 were due to specific diagnoses such as bronchiolitis (26.6%), gastroenteritis (5.7%), poor weight gain (2.3%). The remainder of the URH30 were due to broader causes such as malaise, convulsions, accidents and unspecified illnesses or events (64.9%).

Table 1 shows the distribution of the ten primary predictors amongst the eligible population of 3841 babies by URH30 status. The rate of URH30 was greater in babies of 22–26 weeks gestation, with 72 (15.2%) compared to 238 (10.1%) in 27–31 weeks and 40 (4.0%) in 32–34 week babies (*p* < 0.001). The rate was also higher for those in receipt of nitric oxide (*p =* 0.02) or surfactant (*p* < 0.001), diagnosed with BPD (*p* < 0.001). There were also more URH30 with increasing PMA at discharge (*p* < 0.001), increased discharge weight (*p =* 0.001) and lower levels of breastfeeding (*p =* 0.002). Of the ten additional clinical, mother and socioeconomic predictors, level of birth unit as well as rates of late onset infection and smoking were significantly different according to URH30 status. The cross-tabulation for all additional variables can be seen in Additional file 3.

**Table 1:** Distribution of ten primary characteristics of 3841 eligible babies in the EPIPAGE 2 cohort by 30-day unplanned rehospitalisation (URH30) status. Including missing values. *P* values derived from the chi-squared test

Variables	Total	URH30	URH30 (%) (95% CI)	*p* value
Sex				
Female	1818	152	8.4 (7.1–9.7)	
Male	2001	198	9.9 (8.6–11.2)	0.11
Gestation age (weeks)				
32–34	997	40	4.0 (2.8–5.2)	
27–31	2349	238	10.1 (8.9–11.3)	
22–26	473	72	15.2 (12.0–18.4)	<0.001
Small for gestational age				
Yes	1325	134	10.1 (8.5–11.7)	
No	2494	216	8.7 (7.6–9.8)	0.16
Nitric Oxide				
Yes	163	24	15.0 (10.0–20.0)	
No	3594	324	9.0 (8.1–9.9)	0.02
Surfactant				
Yes	1888	225	11.9 (10.4–13.4)	
No	1885	119	6.3 (5.2–7.4)	<0.001
Early onset neonatal infection				
Yes	609	61	10.0 (7.6–12.4)	
No	3086	275	8.9 (7.9–9.9)	0.43
Bronchopulmonary dysplasia				
None	2915	224	7.7 (6.7–8.7)	
Mild	431	65	15.1 (11.7–18.5)	
Moderate	106	18	17.0 (10.0–24.0)	
Severe	222	30	13.5 (9.0–18.0)	<0.001
Post-menstrual age at discharge (weeks)				
< 36	639	25	3.9 (2.4–5.4)	
36 – <37	960	83	8.6 (6.8–10.4)	
37 – <38	763	70	9.2 (7.2–11.3)	
≥ 38	1442	172	11.9 (10.2–13.6)	<0.001
Discharge weight (grams)				
≤ 2200	706	46	6.5 (4.7–8.3)	
2201–2600	1484	134	9.0 (7.5–10.5)	
2601–3000	977	90	9.2 (7.4–11.0)	
> 3000	579	76	13.1 (10.4–15.9)	0.001
Breastfeeding status				
None	1719	189	11.0 (9.5–12.5)	
Mixed	839	70	8.3 (6.4–10.2)	
Exclusive	1004	72	7.2 (5.6–8.8)	0.002

### Timing of unplanned rehospitalisation over the first 30 days

**Fig. 2:**
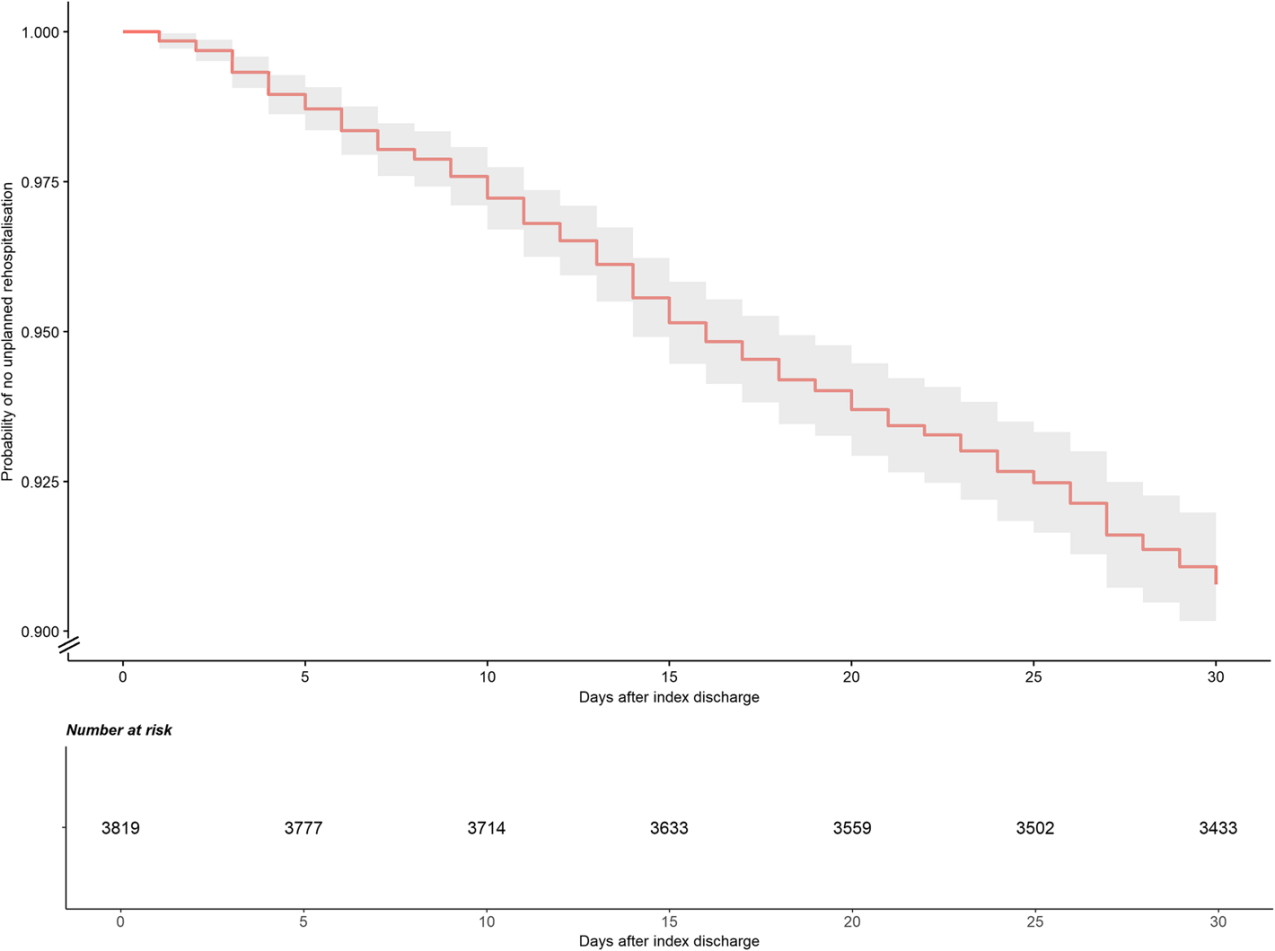
Kaplan-Meier curve with shaded 95% confidence interval and risk table for unplanned rehospitalisation over the first 30 days amongst 3841 eligible babies in the EPIPAGE 2 cohort

The cumulative probability of URH progressed at a relatively consistent rate over the first 30 days following discharge (Fig. 2 and Additional file 4). The probability of URH was 2.8% (95% CI 2.3–3.3) at day 10, 6.3% (95% CI 5.5–7.1) at day 20 and 9.2% (95% CI 8.3–10.1) by day 30.

**Fig. 3:**
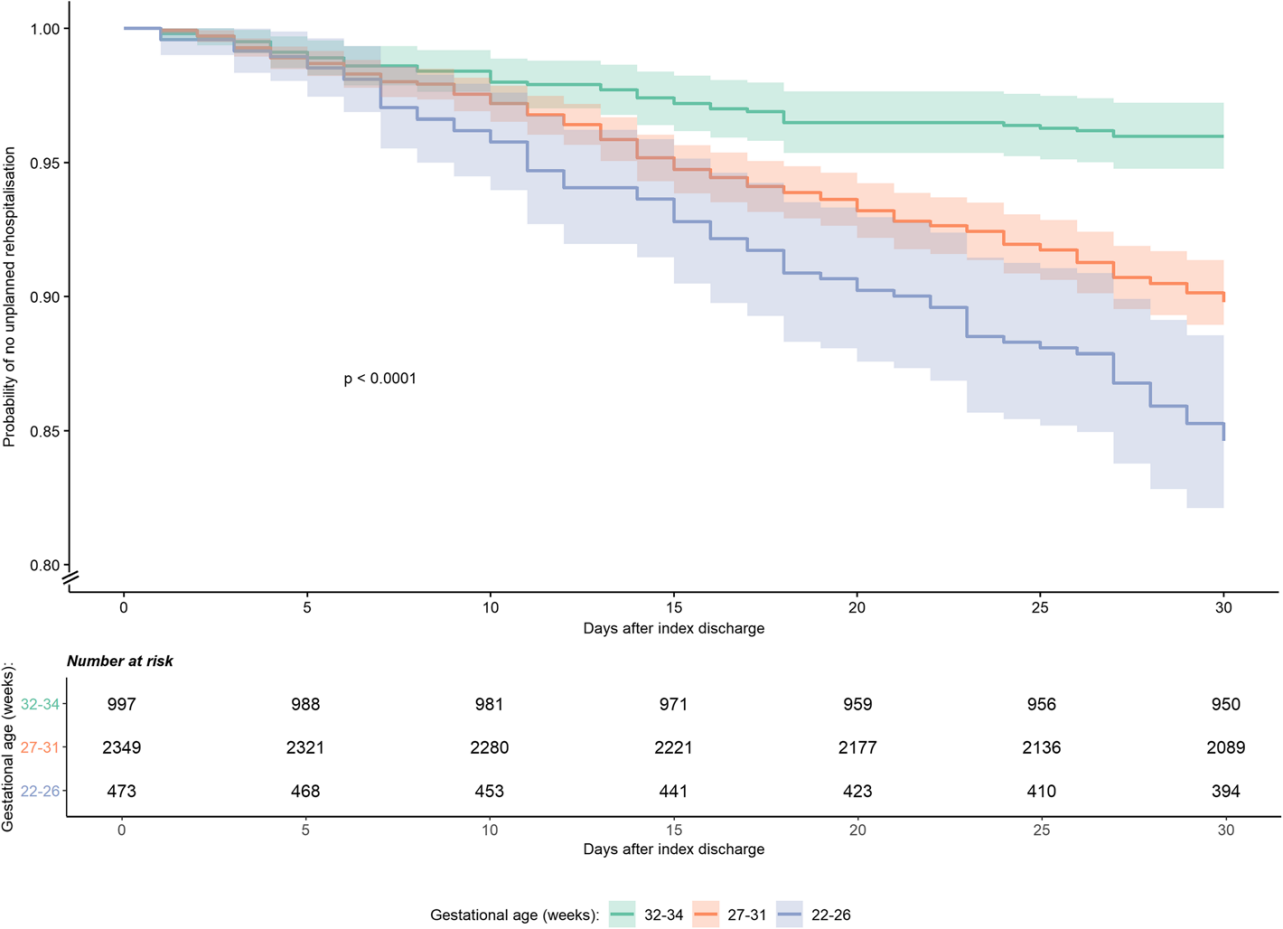
Kaplan-Meier curves with shaded 95% confidence interval and risk table for unplanned rehospitalisation over the first 30 days amongst 3841 eligible babies in the EPIPAGE 2 cohort, by gestational age of babies. *P* value relates to log-rank test, with a null hypothesis that the survival curves are the same

In the three gestational age categories, the probability of URH remained similar initially and began to diverge around day 10. By day 30, the URH probabilities for babies of 22–26, 27–31 and 32–34 weeks gestation were 15.4% (95% CI 12.0–18.6), 10.2% (95% CI 9.0–11.4) and 4.0% (95% CI 2.8–5.2) respectively (Fig. 3 and Additional file 5). Differences between the three Kaplan-Meier curves for the gestational age groups were significant (log-rank test, *p* < 0.001).

### Predictive model performance

Complete-cases were used for the logistic regression model building. Of the 3841 eligible babies, 2707 (70.5%) were complete-cases.

Univariate regression analysis of the ten primary predictor variables shown in Table 2 show gestational age less than 32 weeks, nitric oxide, surfactant, BPD, PMA of 36 weeks or more, discharge weight greater than 3000 g and breastfeeding status were all independently associated with URH30. After adjustment in the multivariate regression predictive model, two variables were found to be significant risk factors (Table 2). These were gestational ages of 22–26 weeks (aOR 1.44 (95% CI 1.18–1.77) and 27–31 weeks (aOR 1.47 (95% CI 1.17–1.84)) compared to 32–34 week babies, and PMA of both 36 to less than 37 weeks (aOR 1.34 (95% CI 1.06–1.70) and 37 to less than 38 weeks (aOR 1.32 (95% CI 1.05–1.65) compared to less than 36 weeks. Results of regression analysis for the 20 predictor model are shown in Additional file 6.

**Table 2:** Unadjusted (uOR) and adjusted odds ratios (aOR) for the ten predictors in the primary predictive logistic regression model for unplanned rehospitalisation within 30 days (URH30) amongst 2707 eligible, complete-case babies in the EPIPAGE 2 cohort

Variable	uOR	95% CI	*p* value	aOR	95% CI	*p* value
Female	0.84	0.65–1.08	0.176	0.92	0.81–1.05	0.22
Gestational age (weeks) (ref. 32–34)						
27–31	2.83	1.82–4.42	<0.001	1.47	1.17–1.84	0.001
22–26	4.88	2.95–8.08	<0.001	1.44	1.18–1.77	<0.001
Small for gestational age	1.11	0.85–1.45	0.440	1.12	0.96–1.30	0.16
Nitric oxide	1.71	1.01–2.92	0.047	1.03	0.92–1.16	0.58
Surfactant	2.10	1.60–2.75	<0.001	1.16	0.99–1.36	0.07
Early onset neonatal infection	1.18	0.85–1.64	0.313	0.99	0.88–1.13	0.91
Bronchopulmonary dysplasia (ref. none)						
Mild	2.26	1.62–3.14	<0.001	1.12	0.98–1.27	0.09
Moderate	2.32	1.30–4.13	0.004	1.05	0.93–1.17	0.45
Severe	1.78	1.12–2.84	0.015	0.98	0.85–1.12	0.73
Post-menstrual age at discharge (weeks) (ref. <36)						
36 - <37	2.22	1.29–3.80	0.004	1.34	1.06–1.70	0.02
37 - <38	2.48	1.43–4.30	0.001	1.32	1.05–1.65	0.02
≥ 38	3.04	1.83–5.05	<0.001	1.29	0.97–1.72	0.08
Discharge weight (grams) (ref. 2,201–2600)						
≤2200	0.79	0.53–1.19	0.260	0.91	0.77–1.08	0.28
2601 - 3000	1.22	0.89–1.69	0.220	1.02	0.88–1.19	0.79
> 3000	1.60	1.13–2.28	0.008	1.05	0.90–1.23	0.52
Breastfeeding status (ref. none)						
Mixed	0.79	0.57–1.10	0.160	0.95	0.82–1.09	0.47
Exclusive	0.69	0.50–0.94	0.019	0.88	0.76–1.02	0.10

### Discrimination

**Fig. 4:**
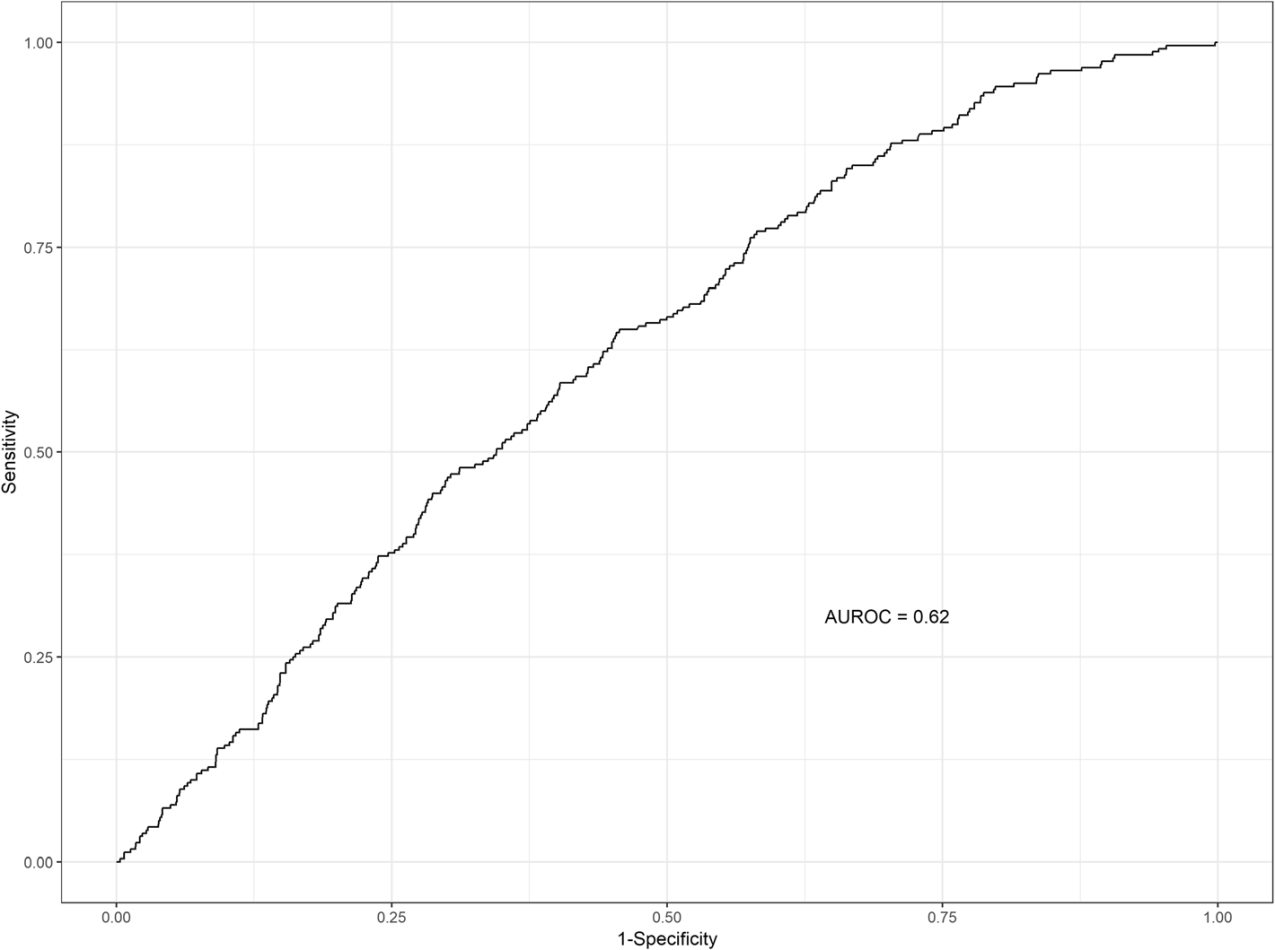
Receiver operating characteristic curve (ROC) of cross-validated predictions and corresponding area under the curve (AUROC) for the ten predictor model for unplanned rehospitalisation within 30 days developed on 2707 eligible, complete-case babies in the EPIPAGE 2 cohort

The discriminatory performance of all three models was similar. The model containing gestational age alone gave an AUROC of 0.60 (95% CI 0.57–0.62). For the ten predictor model, the AUROC across the ten cross-validated sets was 0.62 (95% CI 0.59–0.65). Figure 4 shows the cross-validated ROC resulting from the predictions on the ten test sets. At a classification threshold of 0.08, sensitivity and specificity for the ten predictor model were 0.77 and 0.42 respectively. Tjur’s coefficient was 0.019.

A more complex model, containing twenty predictors gave an AUROC of 0.62 (95% CI 0.58–0.72). Full discrimination performance results for each of the three predictive models can be seen in Table 3. The inclusion of continuous versions of variables such as gestational age and birth weight (in place of their categorised equivalents) did not improve the predictive power of the models.

**Table 3:** Predictive performance measures for logistic regression models constructed on 2707 eligible, complete-case babies in the EPIPAGE 2 cohort and validated using 10-fold cross-validation

Model	AUROC	95% CI	Sensitivity	Specificity	Tjur’s Coefficient	Hosmer-Lemeshow
One predictor	0.60	0.57–0.62	0.91	0.23	0.015	0.003
Ten predictor	0.62	0.59–0.65	0.77	0.42	0.019	<0.001
20 predictor	0.62	0.58–0.72	0.72	0.46	0.020	<0.001
Ten predictor (imputed data)^a^	0.63 (IQR 0.004)	–	0.75 (IQR 0.01)	0.45 (IQR 0.01)	0.019 (IQR 0.001)	<0.001

^a^Performance measured over 20 imputed data sets and measures reported as median and inter-quartile range (IQR)

### Calibration

**Fig. 5:**
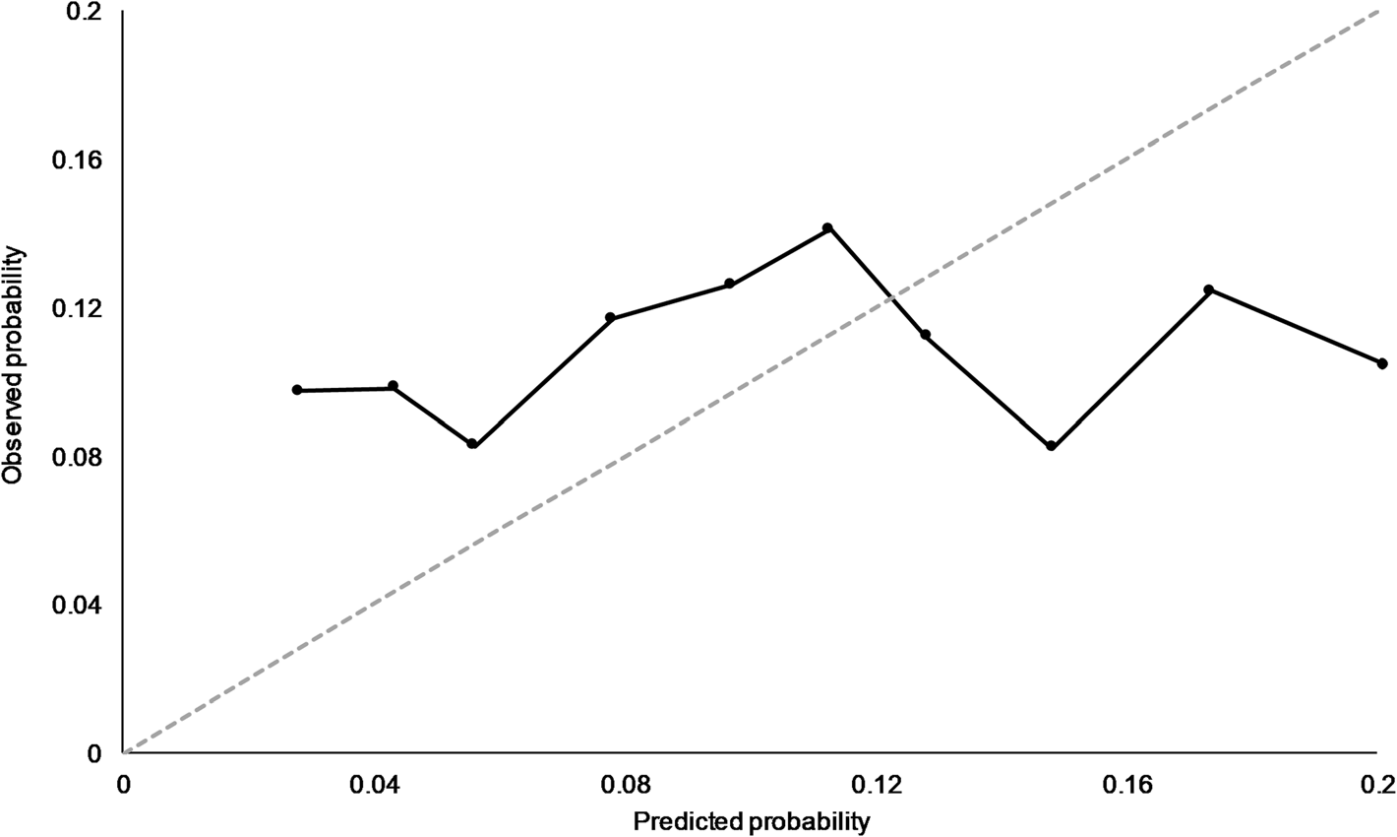
Calibration curve for the cross-validated ten predictor model comparing the observed probability of unplanned rehospitalisation within 30 days with predicted probability across risk deciles developed on 2707 eligible, complete-case babies in the EPIPAGE 2 cohort. Hosmer-Lemeshow test *p* < 0.001

Figure 5 and Table 3 show the calibration curve and *p* value derived from the Hosmer-Lemeshow test for the ten predictor model. The test offered sufficient evidence to reject the null hypothesis that, across risk deciles, actual and observed URH30 event counts were similar to predicted counts. Full discrimination performance results for each of the three predictive models can be seen in Table 3.

### Sensitivity to missing data

A total of 1134 (29.5%) had missing data on explanatory variables. Rates of missingness by variable are shown in Additional file 2. The ten predictor model built with multiply imputed data gave a median AUROC of 0.63 (IQR 0.004), sensitivity of 0.75 (IQR 0.01), specificity of 0.45 (IQR 0.01) and a Tjur’s statistic of 0.019 (IQR 0.001) (Table 3).

## Discussion

Using data from a prospective, population-based cohort study of 3841 newborns of 22–34 weeks gestation (EPIPAGE 2), we found that the overall risk of early unplanned rehospitalisation within 30 days of discharge was approximately 9%. The timing of rehospitalisations during the 30-day period had a fairly uniform distribution with an approximately linear progression of rehospitalisation risk during the period. Compared to babies of 32–34 weeks gestation, those of 22–26 and 27–31 weeks gestation had a statistically important increased probability of URH within 30 days. Lower gestational age and increased PMA at discharge were associated with URH30 in the ten predictor model. The association with increased PMA at discharge might reflect difficulties at home, leading to delayed discharge and a subsequent increased risk of rehospitalisation when a baby is finally discharged. A predictive model based on ten clinically important variables chosen after a review of the literature and input from expert clinicians, as well as models containing a more extensive set of predictors, showed relatively poor discrimination and low indices of predictive ability. Moreover, the added value of other variables compared to a predictive model based on gestational age alone was quite limited. Given the key role gestational age plays in determining physiological immaturity, and that lower gestational age is a recognised risk factor for rehospitalisation, this finding was not unexpected [23, 24, 52]. The limited predictive ability of our models is in line with the literature on predictive models for rehospitalisation in adults, in which a majority fail to achieve clinically useful performance [53].

### Strengths and limitations

Using data from a large population-based study these results provide useful insight into the probability and timing of early unplanned rehospitalisation in preterms, especially amongst less extensively studied extremely and very preterm babies. This study is, to the best of our knowledge, the first validated predictive model for early rehospitalisation in preterm babies. The range of information collected meant that established risk factors, and more unique variables, were available for analysis and consideration in predictive modelling. The wide range of available variables and subsequent selection in consultation with clinical experts increased the likelihood that clinically relevant predictors were included in the models. Our choice of a 30-day follow-up period for our outcome was based on its established use in the literature and as a quality measure in health systems such as the UK National Health Service [54] and Medicare and Medicaid Services in the United States [55]. The period is considered appropriate as it is short enough to limit the influence of factors outside the immediate control of clinicians, thus potentially making such rehospitalisations more amenable to preventive adjustments in treatment or discharge decisions for example.

We chose to focus on unplanned rehospitalisation as planned causes are less likely to be preventable. We acknowledge the decision to classify all surgical rehospitalisations as planned may have led to the exclusion of surgical interventions that were unplanned. The construction of our outcome also relied upon mothers recalling the date and cause of their baby’s three longest rehospitalisations. Though bias was minimised through verification using the child’s hand-held record, some errors still persist. It is also possible that shorter rehospitalisation for transient illnesses may be under represented in our sample. It is difficult to say whether these limitations might have impacted the predictive power of our models. Furthermore, we excluded 22 babies who died between discharge and one-year follow-up. It was not possible to establish the exact dates of these deaths and whether they occurred within 30 days of discharge. However, although only a very small proportion of the babies discharged home alive died (0.5%), excluding them might have introduced bias: babies that died were more likely to have severe illness, and thus more likely to have been rehospitalised within 30 days of discharge.

Unplanned rehospitalisation within 30 days in this study was relatively infrequent and is in line with much of the literature on early rehospitalisations in preterm babies [19, 33, 56]. Building predictive models on infrequent outcomes presents difficulties for classification and the default 0.50 classification threshold can be inappropriate [44, 57]. Regression models built using infrequent event data can produce negatively biased intercepts with underestimated predicted probabilities in the direction of the majority outcome [58–60]. To address this, we used a prevalence dependent threshold of 0.08 to optimise the false positive and true positive rates, as recommended [44–46].

We acknowledge that performance measures derived through cross-validation are inferior to those derived from external validation methods using independent data. However, obtaining external data can often be challenging and cross-validation represents a powerful alternative, especially compared to techniques such as split-sampling which can significantly reduce the size of the training and test samples. Future model building might require the identification of a wider range of predictors. Alternative machine learning techniques, for example penalised regression or random forest analysis, would allow for the consideration of many more variables whilst limiting over-fitting.

## Conclusion

We conclude that early, unplanned rehospitalisations of very preterm babies affect approximately 9% of our population. Over the 30 days following initial discharge there was a generally linear progression of rehospitalisation risk. Ultimately, predicting unplanned rehospitalisation with a range of clinical, maternal, and socioeconomic predictors proved challenging in our study. Given the cost and burden associated with rehospitalisations, it remains important that we maintain efforts to better understand and predict such outcomes. This may in turn facilitate the implementation of strategies to prevent unplanned rehospitalisations in preterm babies.

## Supplementary information

**Additional file 1.** Forty-eight potential predictors of unplanned rehospitalisation in preterm babies considered for inclusion in predictive models in consultation with field experts. All predictors were derived from the EPIPAGE 2 study.

**Additional file 2.** Thirty variables included as predictors in multivariate imputation by chained equations for missing data amongst 3841 eligible babies in the EPIPAGE 2 cohort.

**Additional file 3.** Distribution of ten additional predictor variables amongst 3841 eligible babies in the EPIPAGE 2 cohort by 30-day unplanned rehospitalisation (URH30) status. Including missing values. *P*-values derived from the chi-squared test for categorical variables and Kruskal-Wallis test for continuous.

**Additional file 4.** Cumulative probability of no unplanned rehospitalisation (URH) (and the inverse) over the first 30 days following discharge from birth hospitalisation, amongst 3841 eligible babies in the EPIPAGE 2 cohort. Derived via Kaplan-Meier analysis.

**Additional file 5.** Cumulative probability of no unplanned rehospitalisation (URH) (and the inverse) over the first 30-days following discharge from birth hospitalisation by gestational age (GA) category, amongst 3841 eligible babies in the EPIPAGE 2 cohort. Derived via Kaplan-Meier analysis.

**Additional file 6.** Unadjusted (uOR) and adjusted odds ratios (aOR) for the 20 predictor predictive logistic regression model for unplanned rehospitalisation within 30-days (URH30), amongst 2707 eligible complete-case babies in the EPIPAGE 2 cohort.

## Data Availability

Data used in the current study are not publicly available as they contain confidential information but are available from the Scientific Group of the EPIPAGE 2 study for researchers who meet the criteria for access to confidential data on reasonable request.

## Abbreviations

aOR: Adjusted odds ratio
AUROC: Area under the receiver-operating characteristic curve
BPD: Bronchopulmonary dysplasia
CI: Confidence interval
EPIPAGE 2: Etude épidémiologique sur les petits âges gestationnels /Epidemiological study on small gestational ages
IQR: Interquartile range
IVH: Intraventricular haemorrhage
PMA: Post-menstrual age at discharge
ROC: Receiver-operating characteristic curve
SGA: Small for gestational age
uOR: Unadjusted odds ratio
URH: Unplanned rehospitalisation after index discharge from birth hospitalisation
URH30: Unplanned rehospitalisation within 30 days of index discharge from birth hospitalisation
