# Supplementary material for: Assessing the risk of early unplanned rehospitalisation in preterm babies: EPIPAGE 2 study
Source: BMC Pediatr. 2019 Nov 21;19:451. doi: 10.1186/s12887-019-1827-6 (PMC6870221; doi:10.1186/s12887-019-1827-6)
Supplement: Supplementary file 1 — Additional file 1. Forty-eight potential predictors of unplanned rehospitalisation in preterm babies considered for inclusion in predictive models in consultation with field experts. All predictors were derived from the EPIPAGE 2 study. [file 12887_2019_1827_MOESM1_ESM.docx]

| Variable | Type |
| --- | --- |
| Sex | Binary |
| Gestational age (weeks) | Categorical |
| Birth weight (grams) | Categorical |
| Small for gestational age | Binary |
| Mode of delivery | Categorical |
| Type of birth (single, twin, triple, quad) | Categorical |
| Congenital abnormality | Binary |
| Neonatal morbidity | Binary |
| Early onset neonatal infection (within ≤72h life) | Binary |
| Late onset neonatal infection (>72h of life) | Binary |
| Bronchopulmonary dysplasia | Categorical |
| Respiratory event | Binary |
| Necrotising entercolitis | Binary |
| Intraventricular hemorrhage (Stage 3 IVH or IPH) | Binary |
| Surfactant | Binary |
| Nitric oxide | Binary |
| Palivizumb | Binary |
| Days of mechanical ventilation | Continuous |
| Gastroespoghageal reflux treatment | Binary |
| Sedative and/or analgesic of class I or II | Binary |
| Sedative and/or analgesic of class III | Binary |
| Vaccinations initiated during birth hospitalisation | Binary |
| Level of birth establishment | Categorical |
| Number of establishments in which baby was hospitalised | Continuous |
| Baby's age at discharge (days) | Continuous |
| Weight at discharge (grams) | Continuous |
| Breastfeeding at discharge | Categorical |
| Left hospital with monitoring/care equipment (O_2_, gastroespohageal reflux, apnea/bradycardia or feeding tube) | Binary |
| Nasogastric feeding at discharge | Binary |
| First follow up meeting with the baby has been fixed | Binary |
| Mother's age (years) | Continuous |
| Mother's birth place | Binary |
| Mother lives in a couple | Binary |
| Family socioeconomic status | Categorical |
| Mother's employment status | Binary |
| Monthly household income | Categorical |
| Mother's social cover | Categorical |
| Mother smoking during pregnancy | Binary |
| Skin to skin (within 0-7 days of birth) | Binary |
| Number of previous pregnancies | Continuous |
| Mother has hypertension and/or diabetes outside pregnancy | Binary |
| Mother has alcohol or drug addiction | Binary |
| Mother has history of psychiatric disorders | Binary |
| Mother has other chronic pathologies | Binary |
| First pregnancy consultation before 15 weeks | Binary |
| Mother taught signs of discomfort and how to calm baby | Binary |
| Breastfeeding counselling available | Categorical |
| Mother felt supported by care team during birth hospitalisation | Binary |

Table 1: Forty-eight potential predictors of unplanned rehospitalisation amongst preterm babies considered for inclusion in predictive models in consultation with field experts. All predictors were variables derived from the EPIPAGE 2 study.
