# Supplementary material for: Assessing the risk of early unplanned rehospitalisation in preterm babies: EPIPAGE 2 study
Source: BMC Pediatr. 2019 Nov 21;19:451. doi: 10.1186/s12887-019-1827-6 (PMC6870221; doi:10.1186/s12887-019-1827-6)
Supplement: Supplementary file 2 — Additional file 2. Thirty variables included as predictors in multivariate imputation by chained equations for missing data amongst 3841 eligible babies in the EPIPAGE 2 cohort. [file 12887_2019_1827_MOESM2_ESM.docx]

| Variable | Variable type | Proportion missing | Imputation method |
| --- | --- | --- | --- |
| Sex | Binary | Zero | - |
| Gestational age (weeks) | Continuous | Zero | - |
| Birth weight (grams) | Continuous | Zero | - |
| Small for gestational age | Binary | Zero | - |
| Mode of delivery | Categorical | 0.01 | Polytomous regression |
| Type of birth (single, twin, triple, quad) | Categorical | Zero | - |
| Congenital abnormalities | Binary | Zero | - |
| Early onset neonatal infection (within ≤72h life) | Binary | 0.03 | Logistic regression |
| Late onset neonatal infection (>72h of life) | Binary | 0.06 | Logistic regression |
| Bronchopulmonary dysplasia stage | Categorical | 0.04 | Proportional odds model |
| Necrotising entercolitis | Binary | 0.02 | Logistic regression |
| Intraventricular hemorrhage (Stage 3 IVH or IPH) | Binary | 0.06 | Logistic regression |
| Surfactant | Binary | 0.01 | Logistic regression |
| Nitric oxide | Binary | 0.02 | Logistic regression |
| Level of birth establishment | Categorical | Zero | - |
| Nasogastric feeding at discharge | Binary | 0.03 | Logistic regression |
| Days of mechanical ventilation | Continuous | 0.04 | Predictive mean matching |
| Gastroesophageal reflux treatment | Binary | 0.01 | Logistic regression |
| Age at discharge (days) | Continuous | Zero | - |
| Discharge weight (grams) | Continuous | 0.02 | Predictive mean matching |
| Breastfeeding at discharge | Categorical | 0.07 | Proportional odds model |
| Mother's age (years) | Continuous | Zero | - |
| Mother's birth place | Categorical | Zero | - |
| Mother lives in couple | Binary | 0.05 | Logistic regression |
| Family socioeconomic status | Categorical | 0.04 | Proportional odds model |
| Number of previous pregnancies | Continuous | Zero | - |
| Mother smoking during pregnancy | Binary | 0.03 | Logistic regression |
| Days between discharge and hospitalisation 1 | Continuous | 0.01 | Predictive mean matching |
| Days between discharge and hospitalisation 2 | Continuous | Zero | - |
| Days between discharge and hospitalisation 3 | Continuous | Zero | - |

Table 2: Thirty variables included as predictors in multivariate imputation by chained equations of missing data amongst 3,841 eligible babies in the EPIPAGE 2 cohort.
