# Supplementary material for: Assessing the risk of early unplanned rehospitalisation in preterm babies: EPIPAGE 2 study
Source: BMC Pediatr. 2019 Nov 21;19:451. doi: 10.1186/s12887-019-1827-6 (PMC6870221; doi:10.1186/s12887-019-1827-6)
Supplement: Supplementary file 3 — Additional file 3. Distribution of ten additional predictor variables amongst 3841 eligible babies in the EPIPAGE 2 cohort by 30-day unplanned rehospitalisation (URH30) status. Including missing values. P-values derived from the chi-squared test for categorical variables and Kruskal-Wallis test for continuous. [file 12887_2019_1827_MOESM3_ESM.docx]

| Variable | Total | URH30 | URH30 (%) (95% CI) | *P value* |
| --- | --- | --- | --- | --- |
| Multiple pregnancy |  |  |  |  |
| Yes | 1308 | 115 | 8.8 (7.3-10.3) |  |
| No | 2511 | 235 | 9.4 (8.2-10.5) | 0.61 |
| Level of birth unit |  |  |  |  |
| 1 | 113 | 11 | 10 (4-15) |  |
| 2a | 327 | 18 | 5.5 (3.0-8.0) |  |
| 2b | 437 | 26 | 5.9 (3.7-8.1) |  |
| 3 | 2942 | 295 | 10 (8.9-11.1) | 0.004 |
| Congenital abnormality |  |  |  |  |
| Yes | 392 | 41 | 10.5 (7.5-13.5) |  |
| No | 3412 | 307 | 9 (8.0-10.0) | 0.39 |
| Late onset neonatal infection |  |  |  |  |
| Yes | 1243 | 154 | 12.4 (10.6-14.2) |  |
| No | 2330 | 174 | 7.5 (6.4-8.6) | <0.001 |
| Necrotising entercolitis |  |  |  |  |
| Yes | 116 | 17 | 15 (8-21) |  |
| No | 3645 | 328 | 9 (8.1-9.9) | 0.06 |
| Intraventricular hemorrhage |  |  |  |  |
| Yes | 104 | 9 | 9 (3-14) |  |
| No | 3481 | 333 | 9.6 (8.6-10.6) | 0.89 |
| Mother's age (years) (IQR) | 29 (7) / 30 (8) | - | - | 0.05 |
| Mother born outside France |  |  |  |  |
| Yes | 793 | 67 | 8.4 (6.5-10.3) |  |
| No | 3014 | 283 | 9.4 (8.4-10.4) | 0.46 |
| Family socioeconomic status |  |  |  |  |
| Professional | 862 | 83 | 9.6 (7.6-11.6) |  |
| Intermediate | 807 | 64 | 7.9 (6.0-9.8) | 0.71 |
| Administrative, public service, sdfsdself-employed, student | 996 | 99 | 9.9 (8.1-11.8) |  |
| Shop assistant, service worker | 512 | 47 | 9.2 (6.7-11.7) |  |
| Manual worker | 393 | 38 | 9.7 (6.8-12.6) |  |
| No profession | 85 | 6 | 7 (2-13) | 0.71 |
| Smoking during pregnancy |  |  |  |  |
| Yes | 760 | 89 | 11.7 (9.4-14.0) |  |
| No | 2932 | 251 | 8.6 (7.6-9.6) | 0.009 |

Table 3: Distribution of ten additional predictor variables amongst 3,841eligible babies in the EPIPAGE 2 cohort by 30-day unplanned rehospitalisation (URH30) status. Including missing values. P-values derived from the chi-squared test for categorical variables and Kruskal-Wallis test for continuous.
