# Supplementary material for: Assessing the risk of early unplanned rehospitalisation in preterm babies: EPIPAGE 2 study
Source: BMC Pediatr. 2019 Nov 21;19:451. doi: 10.1186/s12887-019-1827-6 (PMC6870221; doi:10.1186/s12887-019-1827-6)
Supplement: Supplementary file 4 — Additional file 4. Cumulative probability of no unplanned rehospitalisation (URH) (and the inverse) over the first 30 days following discharge from birth hospitalisation, amongst 3841 eligible babies in the EPIPAGE 2 cohort. Derived via Kaplan-Meier analysis. [file 12887_2019_1827_MOESM4_ESM.docx]

| Day | N. at risk | N. URH | No URH prob. | 95% LCL | 95% UCL | URH prob. | 95% LCL | 95% UCL |
| --- | --- | --- | --- | --- | --- | --- | --- | --- |
| 0 | 3819 | 0 | 1.000 | 1.000 | 1.000 | 0.000 | 0.000 | 0.000 |
| 1 | 3819 | 6 | 0.998 | 0.997 | 1.000 | 0.002 | 0.000 | 0.003 |
| 2 | 3812 | 6 | 0.997 | 0.995 | 0.999 | 0.003 | 0.001 | 0.005 |
| 3 | 3806 | 14 | 0.993 | 0.991 | 0.996 | 0.007 | 0.004 | 0.009 |
| 4 | 3792 | 14 | 0.990 | 0.986 | 0.993 | 0.011 | 0.007 | 0.014 |
| 5 | 3777 | 9 | 0.987 | 0.984 | 0.991 | 0.013 | 0.009 | 0.016 |
| 6 | 3766 | 14 | 0.984 | 0.980 | 0.988 | 0.017 | 0.013 | 0.021 |
| 7 | 3749 | 12 | 0.980 | 0.976 | 0.985 | 0.020 | 0.015 | 0.024 |
| 8 | 3736 | 6 | 0.979 | 0.974 | 0.983 | 0.021 | 0.017 | 0.026 |
| 9 | 3730 | 11 | 0.976 | 0.971 | 0.981 | 0.024 | 0.019 | 0.029 |
| 10 | 3714 | 14 | 0.972 | 0.967 | 0.977 | 0.028 | 0.023 | 0.033 |
| 11 | 3700 | 16 | 0.968 | 0.962 | 0.974 | 0.032 | 0.026 | 0.038 |
| 12 | 3684 | 11 | 0.965 | 0.959 | 0.971 | 0.035 | 0.029 | 0.041 |
| 13 | 3673 | 15 | 0.961 | 0.955 | 0.967 | 0.039 | 0.033 | 0.045 |
| 14 | 3656 | 21 | 0.956 | 0.949 | 0.962 | 0.044 | 0.038 | 0.051 |
| 15 | 3633 | 16 | 0.951 | 0.945 | 0.958 | 0.049 | 0.042 | 0.055 |
| 16 | 3614 | 12 | 0.948 | 0.941 | 0.955 | 0.052 | 0.045 | 0.059 |
| 17 | 3600 | 11 | 0.945 | 0.938 | 0.953 | 0.055 | 0.047 | 0.062 |
| 18 | 3586 | 13 | 0.942 | 0.935 | 0.949 | 0.058 | 0.051 | 0.065 |
| 19 | 3573 | 7 | 0.940 | 0.933 | 0.948 | 0.060 | 0.052 | 0.067 |
| 20 | 3559 | 12 | 0.937 | 0.929 | 0.945 | 0.063 | 0.055 | 0.071 |
| 21 | 3547 | 10 | 0.934 | 0.927 | 0.942 | 0.066 | 0.058 | 0.074 |
| 22 | 3535 | 6 | 0.933 | 0.925 | 0.941 | 0.067 | 0.059 | 0.075 |
| 23 | 3528 | 10 | 0.930 | 0.922 | 0.938 | 0.070 | 0.062 | 0.078 |
| 24 | 3518 | 13 | 0.927 | 0.918 | 0.935 | 0.073 | 0.065 | 0.082 |
| 25 | 3502 | 7 | 0.925 | 0.916 | 0.933 | 0.075 | 0.067 | 0.084 |
| 26 | 3495 | 13 | 0.921 | 0.913 | 0.930 | 0.079 | 0.070 | 0.087 |
| 27 | 3477 | 20 | 0.916 | 0.907 | 0.925 | 0.084 | 0.075 | 0.093 |
| 28 | 3454 | 9 | 0.914 | 0.905 | 0.923 | 0.086 | 0.077 | 0.095 |
| 29 | 3445 | 11 | 0.911 | 0.902 | 0.920 | 0.089 | 0.080 | 0.098 |
| 30 | 3433 | 11 | 0.908 | 0.899 | 0.917 | 0.092 | 0.083 | 0.101 |

Table 4: Cumulative probability of no unplanned rehospitalisation (URH) (and the inverse) over the first 30 days following discharge from birth hospitalisation amongst 3,841eligible babies in the EPIPAGE 2 cohort. Derived via Kaplan-Meier analysis.
