# Supplementary material for: Assessing the risk of early unplanned rehospitalisation in preterm babies: EPIPAGE 2 study
Source: BMC Pediatr. 2019 Nov 21;19:451. doi: 10.1186/s12887-019-1827-6 (PMC6870221; doi:10.1186/s12887-019-1827-6)
Supplement: Supplementary file 5 — Additional file 5. Cumulative probability of no unplanned rehospitalisation (URH) (and the inverse) over the first 30-days following discharge from birth hospitalisation by gestational age (GA) category, amongst 3841 eligible babies in the EPIPAGE 2 cohort. Derived via Kaplan-Meier analysis. [file 12887_2019_1827_MOESM5_ESM.docx]

| GA | Day | N. at risk | N. URH | No URH prob. | 95% LCL | 95% UCL | URH prob. | 95% LCL | 95% UCL |
| --- | --- | --- | --- | --- | --- | --- | --- | --- | --- |
| 32-34 weeks | 0 | 997 | 0 | 1.000 | 1.000 | 1.000 | 0.000 | 0.000 | 0.000 |
|  | 2 | 995 | 3 | 0.997 | 0.994 | 1.000 | 0.003 | 0.000 | 0.006 |
|  | 4 | 992 | 6 | 0.991 | 0.985 | 0.997 | 0.009 | 0.003 | 0.015 |
|  | 6 | 986 | 5 | 0.986 | 0.979 | 0.993 | 0.014 | 0.007 | 0.021 |
|  | 8 | 983 | 2 | 0.984 | 0.976 | 0.992 | 0.016 | 0.008 | 0.024 |
|  | 10 | 981 | 4 | 0.980 | 0.971 | 0.989 | 0.020 | 0.011 | 0.029 |
|  | 12 | 976 | 1 | 0.979 | 0.970 | 0.988 | 0.021 | 0.012 | 0.030 |
|  | 14 | 974 | 5 | 0.974 | 0.964 | 0.984 | 0.026 | 0.016 | 0.036 |
|  | 16 | 969 | 4 | 0.970 | 0.959 | 0.981 | 0.030 | 0.019 | 0.041 |
|  | 18 | 966 | 5 | 0.965 | 0.954 | 0.976 | 0.035 | 0.024 | 0.047 |
|  | 20 | 959 | 0 | 0.965 | 0.954 | 0.976 | 0.035 | 0.024 | 0.047 |
|  | 22 | 958 | 0 | 0.965 | 0.954 | 0.976 | 0.035 | 0.024 | 0.047 |
|  | 24 | 957 | 1 | 0.964 | 0.952 | 0.976 | 0.036 | 0.025 | 0.048 |
|  | 26 | 955 | 2 | 0.962 | 0.950 | 0.974 | 0.038 | 0.026 | 0.050 |
|  | 28 | 951 | 2 | 0.960 | 0.948 | 0.972 | 0.040 | 0.028 | 0.052 |
|  | 30 | 950 | 0 | 0.960 | 0.948 | 0.972 | 0.040 | 0.028 | 0.052 |
| 27-31 weeks | 0 | 2349 | 0 | 1.000 | 1.000 | 1.000 | 0.000 | 0.000 | 0.000 |
|  | 2 | 2346 | 7 | 0.997 | 0.995 | 0.999 | 0.003 | 0.001 | 0.005 |
|  | 4 | 2331 | 19 | 0.989 | 0.985 | 0.993 | 0.011 | 0.007 | 0.015 |
|  | 6 | 2315 | 14 | 0.983 | 0.978 | 0.988 | 0.017 | 0.012 | 0.022 |
|  | 8 | 2295 | 9 | 0.979 | 0.973 | 0.985 | 0.021 | 0.015 | 0.027 |
|  | 10 | 2280 | 17 | 0.972 | 0.965 | 0.979 | 0.028 | 0.021 | 0.035 |
|  | 12 | 2262 | 18 | 0.964 | 0.957 | 0.972 | 0.036 | 0.028 | 0.043 |
|  | 14 | 2239 | 29 | 0.952 | 0.943 | 0.961 | 0.048 | 0.040 | 0.057 |
|  | 16 | 2209 | 17 | 0.945 | 0.935 | 0.954 | 0.056 | 0.046 | 0.065 |
|  | 18 | 2190 | 13 | 0.939 | 0.929 | 0.949 | 0.061 | 0.051 | 0.071 |
|  | 20 | 2177 | 16 | 0.932 | 0.922 | 0.942 | 0.068 | 0.058 | 0.078 |
|  | 22 | 2158 | 13 | 0.926 | 0.916 | 0.937 | 0.074 | 0.063 | 0.084 |
|  | 24 | 2149 | 16 | 0.920 | 0.909 | 0.931 | 0.081 | 0.069 | 0.091 |
|  | 26 | 2131 | 16 | 0.913 | 0.901 | 0.924 | 0.087 | 0.076 | 0.099 |
|  | 28 | 2102 | 18 | 0.905 | 0.893 | 0.917 | 0.095 | 0.083 | 0.107 |
|  | 30 | 2089 | 16 | 0.898 | 0.886 | 0.910 | 0.102 | 0.090 | 0.114 |
| 22-26 weeks | 0 | 473 | 0 | 1.000 | 1.000 | 1.000 | 0.000 | 0.000 | 0.000 |
|  | 2 | 471 | 2 | 0.996 | 0.990 | 1.000 | 0.004 | 0.000 | 0.010 |
|  | 4 | 469 | 3 | 0.989 | 0.980 | 0.999 | 0.011 | 0.001 | 0.020 |
|  | 6 | 465 | 4 | 0.981 | 0.969 | 0.993 | 0.019 | 0.007 | 0.031 |
|  | 8 | 458 | 7 | 0.966 | 0.950 | 0.983 | 0.034 | 0.017 | 0.050 |
|  | 10 | 453 | 4 | 0.958 | 0.940 | 0.976 | 0.042 | 0.024 | 0.060 |
|  | 12 | 446 | 8 | 0.941 | 0.920 | 0.962 | 0.059 | 0.038 | 0.080 |
|  | 14 | 443 | 2 | 0.936 | 0.915 | 0.959 | 0.064 | 0.041 | 0.085 |
|  | 16 | 436 | 7 | 0.922 | 0.898 | 0.946 | 0.079 | 0.054 | 0.102 |
|  | 18 | 430 | 6 | 0.909 | 0.883 | 0.935 | 0.091 | 0.065 | 0.117 |
|  | 20 | 423 | 3 | 0.902 | 0.876 | 0.930 | 0.098 | 0.071 | 0.124 |
|  | 22 | 419 | 3 | 0.896 | 0.869 | 0.924 | 0.104 | 0.076 | 0.131 |
|  | 24 | 412 | 6 | 0.883 | 0.854 | 0.913 | 0.117 | 0.088 | 0.146 |
|  | 26 | 409 | 2 | 0.879 | 0.850 | 0.909 | 0.121 | 0.091 | 0.150 |
|  | 28 | 401 | 9 | 0.859 | 0.828 | 0.891 | 0.141 | 0.109 | 0.172 |
|  | 30 | 394 | 6 | 0.846 | 0.814 | 0.880 | 0.154 | 0.120 | 0.186 |

Table 5: Cumulative probability of no unplanned rehospitalisation (URH) (and the inverse) over the first 30-days following discharge from birth hospitalisation by gestational age (GA) category amongst 3,841 eligible babies in the EPIPAGE 2 cohort. Derived via Kaplan-Meier analysis.
