# Supplementary material for: Assessing the risk of early unplanned rehospitalisation in preterm babies: EPIPAGE 2 study
Source: BMC Pediatr. 2019 Nov 21;19:451. doi: 10.1186/s12887-019-1827-6 (PMC6870221; doi:10.1186/s12887-019-1827-6)
Supplement: Supplementary file 6 — Additional file 6. Unadjusted (uOR) and adjusted odds ratios (aOR) for the 20 predictor predictive logistic regression model for unplanned rehospitalisation within 30-days (URH30), amongst 2707 eligible complete-case babies in the EPIPAGE 2 cohort. [file 12887_2019_1827_MOESM6_ESM.docx]

| Variable | uOR | 95% CI | *P value* | aOR | 95% CI | *P value* |
| --- | --- | --- | --- | --- | --- | --- |
| Female | 0.84 | 0.65-1.08 | 0.176 | 0.92 | 0.8-1.05 | 0.23 |
| Gestational age (weeks) (ref. 32-34) |  |  |  |  |  |  |
| 27-31 | 2.83 | 1.82-4.42 | <0.001 | 1.44 | 1.14-1.83 | 0.002 |
| 22-26 | 4.88 | 2.95-8.08 | <0.001 | 1.43 | 1.15-1.78 | 0.001 |
| Small for gestational age | 1.11 | 0.85-1.45 | 0.440 | 1.11 | 0.95-1.29 | 0.21 |
| Multiple pregnancy | 0.97 | 0.74-1.27 | 0.821 | 1 | 0.88-1.15 | 0.98 |
| Level of birth unit (ref. level 1) |  |  |  |  |  |  |
| 2a | 0.82 | 0.27-2.44 | 0.715 | 1.04 | 0.78-1.39 | 0.78 |
| 2b | 1.09 | 0.4-2.99 | 0.872 | 1.19 | 0.87-1.63 | 0.28 |
| 3 | 1.51 | 0.6-3.79 | 0.379 | 1.21 | 0.83-1.76 | 0.32 |
| Congenital abnormality | 0.96 | 0.63-1.47 | 0.864 | 0.94 | 0.83-1.08 | 0.40 |
| Early onset neonatal infection | 1.18 | 0.85-1.64 | 0.313 | 0.99 | 0.87-1.13 | 0.93 |
| Late onset neonatal infection | 1.72 | 1.33-2.22 | <0.001 | 1.03 | 0.89-1.2 | 0.69 |
| Bronchopulmonary dysplasia (ref. none) |  |  |  |  |  |  |
| Mild | 2.26 | 1.62-3.14 | <0.001 | 1.11 | 0.97-1.26 | 0.12 |
| Moderate | 2.32 | 1.3-4.13 | 0.004 | 1.04 | 0.93-1.17 | 0.50 |
| Severe | 1.78 | 1.12-2.84 | 0.015 | 0.97 | 0.84-1.12 | 0.70 |
| Necrotising entercolitis | 2.02 | 1.12-3.65 | 0.020 | 1.07 | 0.96-1.19 | 0.23 |
| Intraventricular hemorrhage | 1.00 | 0.48-2.11 | 0.992 | 0.94 | 0.83-1.07 | 0.36 |
| Nitric oxide | 1.71 | 1.01-2.92 | 0.047 | 1.03 | 0.91-1.15 | 0.65 |
| Surfactant | 2.1 | 1.6-2.75 | <0.001 | 1.17 | 0.99-1.37 | 0.06 |
| Post-menstrual age at discharge (weeks) (ref. <36) |  |  |  |  |  |  |
| 36 - <37 | 2.22 | 1.29-3.8 | 0.004 | 1.33 | 1.05-1.69 | 0.02 |
| 37 - <38 | 2.48 | 1.43-4.3 | 0.001 | 1.31 | 1.04-1.64 | 0.02 |
| ≥38 | 3.04 | 1.83-5.05 | <0.001 | 1.28 | 0.96-1.71 | 0.10 |
| Discharge weight (grams) (ref. 2,201-2,600) |  |  |  |  |  |  |
| ≤2,200 | 0.79 | 0.53-1.19 | 0.260 | 0.91 | 0.77-1.08 | 0.28 |
| 2,601 - 3,000 | 1.22 | 0.89-1.69 | 0.220 | 1.02 | 0.88-1.19 | 0.75 |
| >3,000 | 1.6 | 1.13-2.28 | 0.008 | 1.06 | 0.9-1.24 | 0.49 |
| Breastfeeding status (ref. none) |  |  |  |  |  |  |
| Mixed | 0.79 | 0.57-1.1 | 0.160 | 0.96 | 0.83-1.12 | 0.63 |
| Exclusive | 0.69 | 0.5-0.94 | 0.019 | 0.88 | 0.76-1.03 | 0.11 |
| Mother's age (years) | 0.97 | 0.95-1 | 0.027 | 0.87 | 0.76-1.01 | 0.06 |
| Mother born outside France | 0.86 | 0.62-1.19 | 0.357 | 0.95 | 0.82-1.09 | 0.47 |
| Family socioeconomic status (ref. professional) |  |  |  |  |  |  |
| Intermediate | 0.73 | 0.49-1.08 | 0.112 | 0.85 | 0.72-1 | 0.06 |
| Administrative, public service, self-employed, students | 1.13 | 0.8-1.59 | 0.484 | 0.98 | 0.83-1.15 | 0.80 |
| Shop assistants, service workers | 0.87 | 0.56-1.35 | 0.535 | 0.88 | 0.75-1.04 | 0.13 |
| Manual workers | 0.94 | 0.59-1.5 | 0.808 | 0.91 | 0.77-1.06 | 0.21 |
| No profession | 0.74 | 0.29-1.9 | 0.531 | 0.90 | 0.77-1.05 | 0.18 |
| Smoking during pregnancy | 1.39 | 1.03-1.87 | 0.030 | 1.10 | 0.97-1.25 | 0.15 |

Table 6: Unadjusted (uOR) and adjusted odds ratios (aOR) for the twenty predictor predictive logistic regression model for unplanned rehospitalisation within 30-days (URH30) amongst 2,707 eligible complete-case babies in the EPIPAGE 2 cohort.
